# Value of the Hybrid Operating Theater for an Integrated Approach to Diagnosis and Treatment of Pulmonary Nodules in 2019

**DOI:** 10.3389/fsurg.2019.00036

**Published:** 2019-06-27

**Authors:** Priya R. Kothapalli, Moritz C. Wyler von Ballmoos, Ponraj Chinnadurai, Alan B. Lumsden, Mahesh K. Ramchandani

**Affiliations:** ^1^Department of Cardiothoracic Surgery, DeBakey Heart & Vascular Center, Houston Methodist Hospital, Houston, TX, United States; ^2^Weill Cornell Medicine, New York, NY, United States; ^3^Advanced Therapies, Siemens Medical Solutions Inc., Malvern, PA, United States

**Keywords:** VATS (video-assisted thoracic surgery), hybrid OR, minimally invasive thoracic surgery, pulmonary nodule, hybrid localization

## Background

With wide dissemination of computed tomography (CT) geographically and across disciplines, pulmonary nodules are being diagnosed much more commonly. Specifically, the adoption of population-based screening for lung cancer by means of CT has become common place and has drastically increased the volume of lung lesions of indeterminate etiology that require definitive diagnosis and treatment (1, 2). Less invasive alternatives are now available for the diagnosis or treatment of pulmonary nodules, such as percutaneous needle biopsy, navigational bronchoscopy, ablation and radiation therapies. However, the limitations of small tissue samples and risk of loco-regional recurrence continue to favor surgical resection in most cases. Indications for surgical resection include growing lesions, certain radiographic features that are consistent with malignancy, and lesions in populations at high risk due to age, smoking status or history of exposure to other carcinogenic substances. Importantly, a patient's desire to have a definitive diagnosis should also be considered.

Video-assisted thoracic surgery (VATS) has now become widely adopted and is generally considered the standard of care for resection of pulmonary nodules. As a single procedure, surgical resection remains the only modality offering both definitive diagnosis and treatment for pulmonary nodules (3). VATS is significantly less invasive and has tremendous benefits compared to a traditional thoracotomy approach. At the same time, however, VATS has removed the critical element of palpation that provides important intraoperative tactile feedback. As expected, nodules that are small or more proximal in the lung parenchyma tend to be difficult to identify with limited tactile feedback during VATS. Lesions consisting mainly of a ground-glass opacity (GGO) with limited or no solid component are even more challenging, and can be exceedingly difficult to identify within the parenchyma. During surgery these challenges may result in conversion to thoracotomy or wider resections than necessary, with additional loss in lung function and worse outcomes. Several studies have demonstrated that sub-centimeter nodules and those deeper than 5 mm from the visceral pleura result in conversion from VATS to thoracotomy in 12–63% of cases (4, 5).

CT-guided localization of pulmonary nodules by various techniques is now well-established and has been shown to improve overall success and time to complete resection by VATS, reduced incidence of conversion to thoracotomy and wide resections. However, these localization procedures are invasive and have their own complications. Furthermore, placement of markers in spontaneously breathing and non-paralyzed patient is increasingly more difficult and dangerous the closer lesions are to the diaphragm or vital structures, such as the aorta or heart. Several of these challenges can be overcome, especially in the setting of a hybrid operating room (OR). Here we review contemporary methods, risks and benefits of localizing pulmonary lesions prior to resection. Specifically, we address the value of using a hybrid OR with cone-beam CT imaging capabilities to allow for localization and resection of pulmonary nodules to accomplish definitive diagnosis and treatment in one setting. Finally, we provide a perspective of future developments and directions for the field of thoracic surgery leveraging advanced imaging technology.

## Current Techniques for Localization of Pulmonary Nodules Using Computed Tomography

In broad terms, there are two main approaches to localization using either mechanical devices or injectables. These include hookwires, microcoils, dye, collagen, radioactive labeled solutions, or a combination thereof. Hookwires have been utilized at length to mark breast lesions and lend themselves well for use in relatively solid breast tissue. Given the success in this arena, hookwire placement was adopted by interventional radiologists (IR) and thoracic surgeons to also localize lung nodules. Ciriaco et al. studied 151 patients with pulmonary nodules that were smaller than 1 cm and at least 1.5 cm deep to the visceral pleura, of which 1/3 underwent CT-guided wire localization prior to VATS resection. Of these patients, 7.5% had a pneumothorax and 7.5% dislodged the hookwire prior to resection, but the thoracotomy conversion rate was substantially reduced (17 vs. 7.5%) for a number-needed-to-treat of 11 (6). Other groups have demonstrated similar results.

In general, localization can be done in <45 min, is successful in upward of 95% of lesions, substantially reduces the need for conversion to thoracotomy (4.7%), and shortens the time of the VATS procedure (7–9). Development of a pneumothorax is reported in as high as 38% of cases, occasionally requiring a chest tube placement and decompression for tension pneumothorax. Because of the hook design as well as chest wall dynamics with breathing, hookwires are prone to dislodge (10). More concerning is the 10% chance for serious complications reported in some series and trauma to the lung or other organs that are inadvertently penetrated. Massive air embolism from hookwire placement is rare, but has resulted in death (10, 11). This has led to the exploration of options that do not require leaving behind any fixed objects.

Methylene and patent blue, indocyanine green, lipiodol (a radiopaque fat emulsion), as well as collagen and ^99m^Technetium injection for radioactive labeling adjacent to pulmonary nodules have all been trialed extensively (12–14). The complication rates, especially the occurrence of a pneumothorax, tend to be much lower with these injectables (10). Yet, while some groups have been very successful, the limitations of all these approaches are the spillage or diffusion of injectables away from the actual target for resection. These markers can also be difficult to identify intraoperatively, which then necessitates additional mechanical markers (15). Some have therefore advocated to combine hookwires and dye injection for localization of pulmonary nodules (16). Conversely, many interventionalists now use platinum microcoils for localization. These coils, originally designed for endovascular procedures, can be deployed either just adjacent to the target, or such that part of the coils reside slightly above the visceral pleura (pleural marking). Overall, microcoils compare favorably to hookwires with similar localization success, equal or better resection results, but lower complication rates (17, 18). The coils can be placed using a relatively low radiation dose around 5 mSv (19). Because the coils reside within the parenchyma with little or no protrusion above the visceral pleura, intraoperative fluoroscopy can also be used to confirm inclusion of the lesion of interest (9, 20). In a randomized trial, using microcoils compared to no localization significantly increased the success of VATS resection (93 vs. 48%), shortened operative times (37 ± 39 vs. 100 ± 67 min.), and decreased the number of stapler loads being used (3.7 ± 2 vs. 5.9 ± 3) (21).

A meta-analysis of 46 clinical studies comparing hookwire, microcoils, and dye localization concluded that the success rate for marking the target is high (>95%) with all approaches, but also reported the highest complication rates (driven by pneumothorax and dislodgement) with hookwires, followed by dye and then microcoils attesting the latter the best risk-benefit ratio overall (22).

## Benefits and Limitations of the Hybrid Room Approach

Preoperative CT-guided localization of pulmonary nodules has been shown to be reasonably safe, efficient, and effective. However, using separate entities and facilities for localization and resection causes a logistic burden and potential delays. In one study from a high-volume, tertiary care center, the average time from localization to resection was reported at 136 ± 89 min (23). This delay tends to be at the expense of the patient experience primarily. However, in patients with complications from the localization procedure, downstream consequences must be considered. Additionally, dislodgement of the lung markers remains a challenge. Although less common with microcoils, several papers have identified risk factors for dislodgement that are all tied to motion in some form (24, 25). Hence, additional movement associated with breathing pattern and patient transport add to this risk. Indeed, this is one of the main arguments for conducting both procedures in a single setting.

**Figure 1:**
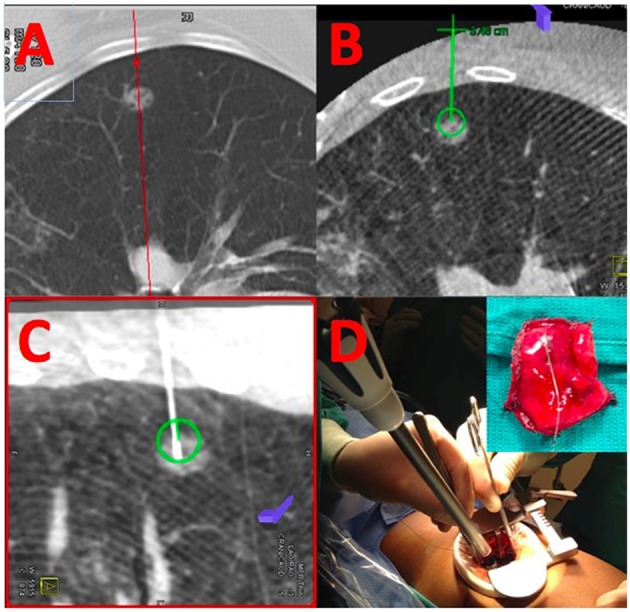
Localization and resection of peripheral GGO. **(A)** Shows a cross-sectional MDCT image of a peripheral ground-glass opacity. The same nodule is visualized by cone-beam CT **(B)** and an appropriate needle trajectory is planned (green line). Using laser-beam guidance, the needle is introduced into the GGO **(C)** using the previously planned trajectory; coils are placed within the lesion, and extending toward the parietal pleura. **(D)** The lesion is resected using a VATS approach using the coils as a landmark for resection.

Integration of real-time on-table imaging and mapping technology is commonplace in cardiology and vascular surgery. This very same technology can also be leveraged for thoracic surgery in a hybrid OR with capabilities to perform cone-beam CT (Figure 1). Gill et al. first reported a prospective clinical trial using this technology with high success rates and low radiation dose (26). Several other reports confirmed the feasibility and success of this one-stop-shop approach (27–29). In a small study comparing standard localization by radiology with hookwires vs. the hybrid room approach dislodgement was substantially reduced (25 vs. 0%) and operative time was essentially equivalent (109 ± 51 vs. 106 ± 42 min.) (30). In a propensity-score matched cohort, the hybrid room approach was also faster (192 vs. 244 min.), albeit the time spent for localization was longer and markers were off target more commonly, likely due to limited experience of the operators (31). Given the success with microcoil placement by navigational bronchoscopy (32), some surgeons have also reported their experience with this approach for placing coils and VATS resection in the hybrid suite (33–35).

Radiation exposure to both the patient and operator also merit consideration. The reported radiation exposure to the patient for localization in the hybrid room is comparable to that using standard CT-guided localization techniques by radiologist (28). An important downside of using the hybrid room approach, of course, is the radiation exposure to the operator, for which data is less readily available. Finally, conducting localization and resection in the hybrid room will increase operating room time. Patients must be positioned and draped in a fashion that allows for free rotation of the cone-beam CT arm around the patient. This usually requires a slightly different set up by the anesthesia team, to provide sufficient space around the patient while allowing for the appropriate monitoring and patient access (28).

## Why Using the Hybrid OR Should Become the Preferred Approach to Management of Pulmonary Nodules

Preoperative localization by IR is commonly used and brings great value to patients that require resection of pulmonary nodules that are otherwise difficult to find intraoperatively. However, this traditional approach requires multiple appointments and trips for the patient and appropriate logistics to coordinate the schedule of the radiologist, surgeon, and OR team. With this approach, the surgeon depends on the availability of an experienced radiologist. On the other hand, surgeons that are trained in using advanced imaging technology, including 3-dimensional image fusion and percutaneous interventions can complete the localization and resection in one sitting. Current evidence suggests that surgeons can perform localization procedures with equivalent precision and time as experienced IR with the added benefit of accomplishing everything in one location (36).

This adds tremendous value to the patient experience and mitigates bad outcomes from complications, such as a pneumothorax during the localization. Further advantages with the hybrid OR approach must also to be considered. Because patients eventually undergo surgical resection, they can be paralyzed and intubated with single lung ventilation even during the localization procedure. They are not moved around and not spontaneously breathing. Eliminating these factors reduces the risk of complications and dislodgement of the marker. In fact, the capabilities of lung isolation, with a controlled persistent inflation state of the lung around the nodules, makes localization easier, more precise, and less risky in a safer environment if complications occur. Although this was shown in a traditional IR setting, the advantage of having partial deflation of the lung, resulting in denser tissue, and no apposition of the lung against the chest wall has previously been demonstrated to improve precision of coil placement and reduce risk of dislodgement (37, 38). Once again, a pneumothorax, even if caused on purpose, is best managed in a controlled environment that is equipped to handle a variety of scenarios. An additional benefit of the hybrid suite is the fluoroscopy capability that extends beyond what can be accomplished with a simple C-arm in a standard OR. In combination with radiopaque markers, the extended fluoroscopy range can be utilized to delineate feasibility of a resection and confirm resection margins before firing a stapler.

## Future Directions and Important Areas of Research for the Use of the Hybrid OR in Thoracic Surgery

Many good arguments can be made to use the hybrid OR for localization and resection of pulmonary nodules. Yet, several important aspects also need further investigation. Current reports have not commented on the feasibility of intraoperative cone-beam CT to address the case of unsuccessful initial resection, requiring re-localization and re-resection in the same sitting. The added benefit of having intraoperative CT imaging capabilities in this rare, but unfortunate, situation would be of interest. Along the same lines, advances in imaging and image processing will eventually enable the use of augmented reality intraoperatively to detect lung nodules, assess resectability, and map resection lines during surgery. This may become particularly helpful in high-risk resection, close to vital structures, and also for cancer resections requiring a complete lymphadenectomy. Initial success with such augmented reality has been demonstrated in a small group of patients, where the investigators used 3-dimensional image-fusion of cone-beam CT in combination with live fluoroscopy to identify and successfully resect pulmonary nodules. The cone-beam CT in these patients was acquired after creating a partial pneumothorax to account for shift in mapping with entrance into the thorax during surgery (39).

Real-time on-table imaging with 3D mapping continues to be a challenge for moving targets, such as the heart and lungs. Several approaches to motion compensation including laser registration and computational modeling are being investigated, and eventually will be able to overcome this limitation. For example, laser registration of the chest wall has been used in radiation oncology to deliver radiation only during a specific point in the respiratory cycle, similar to ECG-gating in cardiac imaging. A further avenue of investigation is the use of other imaging modalities altogether in order to avoid exposure to ionizing radiation. Currently, magnetic resonance imaging is being used increasingly for imaging of the chest. A particular challenge is the temporal resolution when acquiring MRI images, and the need for a prolonged breath-hold, that may be uniquely challenging in patients with underlying lung disease. Furthermore, many of the techniques and tools being used for placement of fiducial markers and resection of pulmonary nodules would have to be re-designed to be MRI compatible.

A major gap in knowledge about the use of the hybrid OR for the management of pulmonary nodules is also the lack of econometric information. Cost considerations often become the primary driving factor. The shorter operating room times with the traditional approach *per se* would certainly discourage the use of the hybrid room approach. For the time being, the hybrid room may be best suited for frail patients and those with friable lung tissue, with both scenarios putting patients at greater risk for complications with localization and sequalae thereof.

Finally, there is an important workforce consideration. The specialty of thoracic surgery has started to blend together with other interventional specialties, such as interventional pulmonology and IR. Undoubtedly, using the hybrid OR with its advanced imaging functions, and real-time on the table imaging and image-fusion requires education and experience above and beyond what is currently part of a typical training program in thoracic surgery. Technological advances will increasingly make less-invasive diagnostic and treatment options available. But going less invasive usually also means relying more on real-time imaging and a distinct skillset to acquire and interpret appropriate imaging information.

## Author Contributions

PK served to gather information and write manuscript with direct conceptual and writing assistance from MW. PC provided important background information, ideas, references, and contributed to writing. AL and MR also directly contributed with content, references, and writing.

### Conflict of Interest Statement

PC is a Senior Staff Scientist employed by Advanced Therapies, Siemens Medical Solutions USA Inc., Malvern, PA. The remaining authors declare that the research was conducted in the absence of any commercial or financial relationships that could be construed as a potential conflict of interest.
